# Defining the boundaries and operational concepts of resilience in the resilience in healthcare research program

**DOI:** 10.1186/s12913-020-05224-3

**Published:** 2020-04-19

**Authors:** Siri Wiig, Karina Aase, Stephen Billett, Carolyn Canfield, Olav Røise, Ove Njå, Veslemøy Guise, Cecilie Haraldseid-Driftland, Eline Ree, Janet E. Anderson, Carl Macrae, on behalf of the RiH-team, Mathilde Bourrier, Siv Hilde Berg, Inger Johanne Bergerød, Lene Schibevaag, Sina Furnes Øyri, Silje Sjøseth, Jane O’Hara, Christophe Edward Kattouw, Foteini Tsandila Kalakou, Signe Berit Bentsen, Tanja Manser, Elisabeth Jeppesen

**Affiliations:** 1 SHARE - Centre for Resilience in Healthcare, Faculty of Health Sciences, University of Stavanger, N-4036 Stavanger, Norway; 2 Griffith University, Brisbane, Queensland Australia; 3 Department of Family Practice, Faculty of Medicine, The University of British Columbia, Vancouver, Canada; 4 Division of Orthopedic Surgery, Oslo University Hospital, Oslo, Norway; 5 Institute of Clinical Medicine, University of Oslo, Oslo, Norway; 6 Department of Safety, Economics and Planning, Faculty of Science and Technology, University of Stavanger, Stavanger, Norway; 7 Florence Nightingale Faculty of Nursing, Midwifery & Palliative Care, King’s College London, London, England; 8 University of Nottingham, Nottingham, England

**Keywords:** Resilience, Healthcare, Adaptive capacity, Change, System perspective, Multi-level approach, Conceptualization

## Abstract

**Background:**

Understanding the resilience of healthcare is critically important. A resilient healthcare system might be expected to consistently deliver high quality care, withstand disruptive events and continually adapt, learn and improve. However, there are many different theories, models and definitions of resilience and most are contested and debated in the literature. Clear and unambiguous conceptual definitions are important for both theoretical and practical considerations of any phenomenon, and resilience is no exception. A large international research programme on Resilience in Healthcare (RiH) is seeking to address these issues in a 5-year study across Norway, England, the Netherlands, Australia, Japan, and Switzerland (2018–2023). The aims of this debate paper are: 1) to identify and select core operational concepts of resilience from the literature in order to consider their contributions, implications, and boundaries for researching resilience in healthcare; and 2) to propose a working definition of healthcare resilience that underpins the international RiH research programme.

**Main text:**

To fulfil these aims, first an overview of three core perspectives or metaphors that underpin theories of resilience are introduced from ecology, engineering and psychology. Second, we present a brief overview of key definitions and approaches to resilience applicable in healthcare. We position our research program with collaborative learning and user involvement as vital prerequisite pillars in our conceptualisation and operationalisation of resilience for maintaining quality of healthcare services. Third, our analysis addresses four core questions that studies of resilience in healthcare need to consider when defining and operationalising resilience. These are: resilience ‘for what’, ‘to what’, ‘of what’, and ‘through what’? Finally, we present our operational definition of resilience.

**Conclusion:**

The RiH research program is exploring resilience as a multi-level phenomenon and considers adaptive capacity to change as a foundation for high quality care. We, therefore, define healthcare resilience as: *the capacity to adapt to challenges and changes at different system levels, to maintain high quality care.* This working definition of resilience is intended to be comprehensible and applicable regardless of the level of analysis or type of system component under investigation.

## Background

### The resilience in healthcare research program (RiH)

The Resilience in Healthcare (RiH) research program is a 5-year study across Norway, England, the Netherlands, Australia, Japan, and Switzerland (2018–2023) [1]. The primary objective of the program is to reform and extend the understanding of quality in healthcare by developing, implementing, and evaluating a theoretical and practical framework of resilience in healthcare. The RiH is a comprehensive research program that models the capacity of healthcare systems and stakeholders to adapt to changes, variations and/or disruptions. The RiH program addresses the following research questions [1]:

1. How can an integrative theoretical framework for RiH be described to understand and improve quality at different system levels?
2. How can involvement of patients and stakeholders in RiH be described and improved?
3. How can RiH be described and improved in different healthcare settings?
4. How can the role of collaborative learning in RiH be described and improved?
5. How can RiH be identified, analysed and compared in different international healthcare settings?

The program has two main phases: i) an explorative phase with screening, synthesis, and validation of results from a sample of existing empirical projects in different healthcare settings (nursing homes, homecare, hospital, prehospital, and regulatory authorities), and ii) an intervention phase with design, implementation, and evaluation of measures to support resilient and adaptive capacities in healthcare quality. Among these activities, we will investigate how resilience is unfolding in different types of healthcare contexts and levels (macro, meso, micro), what kind of mechanisms and triggers are involved, and how different regulatory systems support or hinder resilience in healthcare [1].

The research program provides an important opportunity to bring together and refine many of the contemporary conceptions of resilience in healthcare and then embed and explore this issue through rigorous empirical studies. To do this effectively—both within the ambitious international RiH program and across health services research more broadly—it is important to define a set of initial core operational concepts for resilience that can inform and guide the research program.

### Aims

The aims of this debate paper are to:

1. identify and select core operational concepts of resilience from the resilience literature in order to consider their contributions, implications, and boundaries for researching resilience in healthcare
2. propose a working definition of resilience in healthcare which can underpin the RiH research program, and consider the core questions that may need to be answered to operationalize this definition.

To fulfil these aims, first an overview of three core perspectives that underpin theories of resilience are provided from ecology, engineering and psychology. Second, we present an overview of key definitions and approaches to resilience applicable in healthcare and we propose that collaborative learning and user involvement are vital prerequisite pillars in this conceptualisation and operationalisation of resilience. Third, we present our operational definition of resilience, and then we elaborate on how researchers in the field can use four core questions when they investigate and analyse resilience.

### The importance of defining resilience

There are many different theories, models and definitions of resilience. Many of these are contested and debated in the existing literature [2]. This is perhaps because resilience is primarily a guiding concept used in a range of fields and research traditions, from psychiatry and understanding of individual human response to stress, to societal planning and understanding of response and recovery from large scale disasters, to biology and understanding of resilience in organisms and ecological system functioning [3–5]. Resilience has also become a key concept in safety research. Over the past 10–15 years, ‘resilience engineering’ has become an accepted domain within safety science and has attracted considerable interest by advocating for new ways of understanding work processes in complex adaptive socio-technical systems [2, 6–9].

These diverse theories and models about resilience and the interest in understanding how complex systems continue to operate and deliver services despite stress, disruptions, unforeseen events, and insufficient resources and competence, have also informed and shaped health services research (e.g. [10–17]). It follows, resilient healthcare is a growing research field that seeks to understand and improve system functioning to deliver high quality and safe patient care. Despite the developing interest in resilience across sectors and settings, and a shared use of the terminology of resilience, the definitions vary across disciplinary fields, and the constructs and relationships adopted by them differ widely [2, 18, 19]. This illustrates the importance of carefully defining and conceptualizing the phenomena of resilience.

## Main text

### Core perspectives of resilience: bouncing back, growing, adapting

As noted, the concept of ‘resilience’ is represented in different ways in theories from many and diverse scientific disciplines [2, 4, 9, 20]. Many of these representations provide useful and relevant perspectives that refer to particular mechanisms of response. In simple terms:

- Engineering perspectives of resilience focus on the ability to ‘bounce back’ to some equilibrium state after stress, disruption or surprise. The engineering perspective seeks to understand and strengthen how people adapt and build adaptive capacity into a system or organisation. The engineering perspective is primarily adopted in the safety science.
- Psychological perspectives of resilience focus on the ability of individuals to grow, develop and learn in light of traumas or challenges. In this perspective resilience is an individual psychological capacity often linked to vulnerable groups and how they adapt to cope with adversity, such as abused or neglected children or refugees.
- Ecological perspectives of resilience focus on the ability to adapt and reorganise to maintain core functions and activities. This perspective focuses on how biological systems and communities that face unpredictable and uncertain threats adapt to cope with these and maintain system stability [9, 20].

Despite these variations, some common core mechanisms of resilience have been identified ([3], p. 122) as the ability that individuals, communities, organisational units or larger systems have to return to some ‘normal’ condition or state of functioning after a disruptive event; to cope with pressure and problems by being flexible without compromising system performance; or to adapt to a new normal state, where system functioning is reorganised or enhanced in some way in response to the disruption they face ([3], p. 122). In this view, there is an emphasis on the individuals’, communities’ or organsations’ ability to regain equilibrium in circumstances of changes, or to adapt to new norms, forms, and practices. Either way, the focus is centrally on processes of learning and changing for individuals, communities, and systems, coupled with remaking or transforming individual, interactive, or organisational practices. These mechanisms may help to explain how, in different ways and in different places, healthcare systems are able to deliver high quality care. Any integrative view of resilience [3] in healthcare should be able to accommodate all of these different mechanisms at all levels of a system—while also being clear about the differences between them.

#### A brief overview of resilience definitions and theories

In the following, we briefly synthesise key relevant literature in the field of resilience and describe how resilience has been defined in these literatures. The intention is not to provide a comprehensive review, but to illustrate the different ways that resilience has been defined and the concepts and components previous research has focused on.

Aron Wildavsky [21] is a key figure in safety research and defines resilience as «*the capacity to cope with unanticipated dangers after they have become manifest, learning to bounce back*” (p. 77). A main feature in this conceptualization is that resilience deals with the dangers that have been realized (manifested) and learning emerges as a key element in enabling resilience [21]. Wildavsky [21] emphasises the notions of *active and passive resilience* (Lovins & Lovins [22] – in Wildavsky [21]). Active resilience includes a deliberate effort to improve various abilities to cope with surprises and to use stress as a source of learning, thus actively benefiting from stressful situations. By contrast, passive resilience relates to the ability to bounce back and get back to normal procedures, after an adaptive change has happened, without any further development of skills or systems ([21], p. 98). It follows that the link between resilience and learning is important to explore and understand, such as how learning processes are associated with adaptations in work performance in the face of manifest risks, dangers, and opportunities.

Parts of the resilience literature focus on societal planning and handling major disasters and extreme events. The main concern here is how to design systems able to withstand and respond to major crises such as hurricanes, earthquakes, floods, and terrorist attacks [4]. In this this area both intentional (e.g. terrorist attack) and unintentional threats (e.g. earthquake) are included and Comfort et al. ([4], p. 9) have defined resilience as “*the capacity of a social system (organization, city, or society) to proactively adapt and to recover from disturbances that are perceived within the system to fall outside the range of normal and expected disturbances”.* The social system orientation is important in this area because entire societies, communities, and organisations need to mobilise to adapt and recover from massive unpredicted and large scale disruptions, and return to the normal functioning of a society. Interestingly, the definition includes both proactive identification and prevention of risk as well as recovery from disturbances. That is, resilience can happen before, during or after the occurrence of a disturbance [4]. In addition, in this view the definition of resilience separates individuals and the wider social system, focusing on the latter. This focus on resilience at the level of the social system may be advantageous in the study of organisational and societal responses to large-scale disasters. However, the role of individual actors is also key to understanding how resilience unfolds particularly around the numerous smaller scale disruptions that occur in healthcare [13, 14, 23, 24].

In the safety science literature, the traditional approach focuses on adverse outcomes and ‘find and fix’ solutions, increasingly referred to as a ‘Safety I’ approach. Recently, a ‘Safety II’ perspective has gained interest in healthcare [25, 26], focusing on the processes that support resilient healthcare. Safety II research focuses on learning from why things go right in order to improve safety. Understanding what works well is considered key to understanding what goes wrong [9]. This means researchers should be interested in adaptations made to enable systems to work during stress but also during adaptation to positive changes such as new technology enabling new ways of working. The Safety II approach and Hollnagel et al. ([10], p. xxv) define resilience in healthcare as “*a health care system’s ability to adjust its functioning prior to, during, or following changes and disturbances, so that it can sustain required performance under both expected and unexpected conditions*”. The emphasis is on the system and its adjustments both before and after the disturbances in addition to “changes” that can provide opportunities for the system to transform [26]. According to this definition, resilience is not only related to risk, hazard and danger – it also relates to positive deviances and changes in terms of success, opportunities and disturbances in positive ways [3]. Importantly, resilience and adaptation may not always be beneficial for safety or positive for entire systems: there are circumstances where local adaptations become too extensive and could have negative consequences for the broader system (the ‘tragedy of adaptability’) [27]. An additional trap is that resilience may become constructed in a reductionist and individualistic manner, where the sharp-end operators (e.g. nurses, doctors, patients, next of kin) are expected to handle and compensate for system-caused problems such as ill-designed or under-resourced systems. This can potentially result in burdening the sharp-end practitioners with the responsibility for the resilient performance of a system [9].

This brief conceptual overview illustrates how some of the key definitions of resilience differ, that resilience must thus be understood as a concept with different meanings in different disciplines [4], and that there are also potential traps if resilience is misunderstood or misapplied.

### A brief overview of key constructs in resilience engineering

Resilience engineering provides a conceptualization of resilience that has been most widely applied in healthcare, and so it is useful to explore these concepts in more detail. According to Hollnagel [26] resilience is a characteristic of certain kinds of organisational performance, and the most one can say is that an organization may have the ‘potential’ for resilient performance—the capacity to act in certain ways under certain conditions. To perform resiliently, Hollnagel [10, 26] proposes that organisations need the following underlying resilience ‘potentials’: i) anticipating (knowing what to expect and prepare for), ii) monitoring (knowing what to look for), iii) responding (knowing what to do and adjust to disturbances and changes), and iv) learning (knowing what has happened and learn from experiences). These four resilience potentials are described as interactive functions of an organisational system rather than as individual components, and they constitute a set of integrated functions that depend on and are coupled with each other. An organization draws on these four potentials to perform resiliently, and if it fails in one potential this influences another: they are co-dependent. To exemplify, if an organisation is unable to perform appropriate monitoring functions (e.g. for emerging risks) then it will not be able to execute a proper response (e.g. to manage those new risks). And to monitor, the organization must continuously work on anticipating risk, challenge or change to get a proper information [26]. It is noteworthy that each of these ‘potentials’ are dependent on the actions of a wide range of different actors (e.g. healthcare workers, patients etc.) across a particular system.

Other models have operationalised these ideas and developed models to translate resilience engineering into practice to improve healthcare quality. In the Concepts for Applying Resilience Engineering (CARE) model, Anderson et al. [28] describe the adjustments and adaptations needed to align work-as-imagined (WAI) with work-as-done (WAD). WAI and WAD are key concepts in resilience engineering and highlight differences in the work as intended and described in procedures, regulation and management instructions (WAI), compared to how work in practice often differs from this due to the complexity of the system and local work practices [10, 17]. The CARE model focuses on misalignments between demand and capacity, and how these create the need for adaptations in practice. Mismatches between demand and capacity occur when organisational capacity is insufficient to meet actual demands. The key concepts to analyse are therefore the demands and capacity for work performance, and to understand resilience it is important to be able to identify and characterise these demands and capacities.

A recent review study [29], has explored what key concepts or characteristics are used in resilience in healthcare studies and identified that common resilience characteristics exist across system levels (macro, meso, micro). These relate to anticipation (i.e. looking forward as individual or team to prevent), sensemaking (i.e. perception of something experienced in the current situation in order to adapt), trade-offs (i.e. cognitive as an individual or competing goals as a team), and adaptations (i.e. adjustment to cope with complexity). A key message is that although resilience can be conceptualised in terms of diverse and interconnected activities at different system levels, it can be conceptualized in similar ways at different system levels. This implies the potential for a common terminology for resilience characteristics across system levels, although they may describe somewhat different contents. To illustrate, Berg and Aase [29] discuss both individual cognitive trade-offs and competing goals trade-offs at the team level.

This overview points to the important role of adaptation and the adaptive capacity of individuals and systems; that mechanisms need to be in place to identify and make sense of information during anticipation, monitoring, responding, and learning; and that resilience also involves practical action manifesting in organizational responses to risk, challenge and change. It is also relevant to note that responding to disruptions can also require an understanding of what not to do. Therefore, understanding the decisions and trade-offs made at different system levels is particularly important for understanding resilient performance in research activities.

### Scope and boundaries for resilience in relation to quality, safety and risk

In our RiH research program, we hold that healthcare quality is the key outcome for resilience in healthcare. The healthcare quality concept integrates sub-dimensions of clinical effectiveness, patient safety, patient centeredness, care coordination, efficiency, timeliness, and equity [30–33]. In the literature, resilience is often seen in relation to risk with the aim of preventing risk from manifesting into accidents or harm, and to recover from these [5]. While the safety and risk literature generally limit considerations of resilience to the safety dimension of quality, we propose that the boundary should be wider in healthcare. Doyle et al’s [33] systematic review holds that clinical effectiveness, patient safety and patient experiences, should be considered as a group, and not in isolation. We support this proposition, whilst also proposing that care coordination should be integrated and considered in the resilience conceptualisation, because patients are continuously moving between care levels and settings in modern healthcare systems [34]. The RiH program, therefore, applies four quality dimensions in the operationalization of quality – clinical effectiveness, patient safety, care coordination, and user involvement. User involvement is included, because it focuses on how users are actively involved in healthcare, how they adapt, and therefore how they may provide adaptive capacity that can play a key role in resilience. The related concepts of patient centeredness and patient experience focus more on how healthcare professionals orient their practice around the patient and collect their experiences, and not necessarily on how the patient or user are actively involved in the process.

The RiH program’s emphasis on quality, therefore, means that there is a need to investigate different quality dimensions to understand and operationalise resilience. The five levels of care (i.e. from optimal to poor) described by Vincent and Amalberti [35] may indicate that disruptions and developments over time might have exerted cumulatively positive and negative effects on healthcare quality, a perspective that resilience in healthcare studies need to explore. Until now, this has not been the direction for resilience in healthcare studies, but the cumulative negative effects of suboptimal care, care transitions, and large variations cause increased risk of harm. Furthermore, examining the ability to proactively identify deteriorations in quality of care would be a key resilience mechanism of interest and a key motivation for moving away from retrospective approaches to reducing risk and towards prospectively strengthening systems [28].

### Links amongst resilience, involvement and collaborative learning

User involvement (as described above) and collaborative learning are two prerequisite pillars in our conceptualisation and operationalisation of resilience to maintain quality in healthcare. Collaborative learning through work practice, team work and problem solving is central in quality processes [36, 37]. Previous research and theories, have shown how adaptation is linked to learning in theories of resilience (e.g. [10, 21, 26]), but no systematic emphasis is given to the collaborative element in learning where people in organisations adapt and learn together at work. In healthcare, the patients, users and other stakeholders are also involved in these collaborative learning processes during, for example, diagnosis, treatment, and decision-making at different system levels, e.g. by being part of the care team around one patient [13]; by bringing in relevant learning information about patients or a system; or by making adaptations to ensure the quality of their own services [14]. To understand and promote resilience, we need, therefore, to explore the underlying collaborative learning mechanisms of adaptation, trade-offs, or improvisation happening when people in systems act on disruptions, challenges and changes. Learning occurs continuously in healthcare systems by professionals engaging in clinical work, and by interacting with other co-workers, patients, and stakeholders. Local adaptations, incremental learning and transformation happen in everyday work practices without anyone necessarily noticing [36]. These ideas about learning are consistent with resilience in healthcare in terms of adaptations that people and teams make in their ordinary work. However, the specific links to collaborative learning, learning mechanisms, and learning theory have until now not been clear. So far, the literature has stated that learning is central to resilience as a one of the four resilience potentials [10], but collaborative learning processes have not been investigated in more depth to understand for example what mechanisms actually underpin and support collaborative learning. Integrating resilience with perspectives on collaborative learning is a novel approach. Our view is that resilience as a concept encompasses collaborative learning and therefore, learning mechanisms, tools and theories at the individual and organisational levels need to be more actively integrated into resilience research.

Recent literature indicates that users, patients, families and other stakeholders play a key role in resilience by supporting the healthcare system during times of stress and disruptions [13, 14, 38]. Responses from these important stakeholders contribute to keep the services going in times of peaks, understaffing, and during discontinuity in e.g. care transitions. Patients and family members act as knowledge-brokers between care levels and service providers [14], and next of kin support co-creation of resilience in several areas in cancer care [13]. Next of kin complement healthcare professionals’ resilient performance by their unique insights about the patients and the system, and their respective responses to handling disruptions. When healthcare professionals and the system accept patients’ or next to kin’s information and contribution as important for giving care, resilience can be supported. Consistent with this growing body of literature, we propose that resilience in healthcare cannot be conceptualised or explored without a clear understanding of the role of patients, users, families and other stakeholders in creating or co-creating resilience [23, 24, 39].

### A proposed definition of resilience in healthcare

Drawing on the various concepts, approaches and models of resilience across multiple literatures the RiH program considers resilience in healthcare as the diverse capacities of a healthcare system that allow it to maintain the delivery of high quality care during and after events that challenge, change or disrupt its activities, by engaging people in collaborative and coordinated processes that adapt, enhance or reorganise system functioning in response to those events. Our conceptualisation of resilience can be elaborated in the following bullet points:

- It focuses on maintaining a high-quality healthcare system, incorporating but not limiting it to the handling of safety and risk, and not specifying how ‘high quality healthcare’ should itself be defined and measured;
- It considers resilience as a set of capacities at individual, team and system level that permit high quality to be maintained through adaptation, enhancement and reorganisation – so ‘high quality’ can be a continually moving (and improving) target;
- It is focused on events that may provide challenges, changes or disruptions to the delivery of care – which may include the introduction of new technologies or innovative work practices, challenges in terms of funding or the emergence of new medical conditions, or disruptions in terms of serious unexpected events and stressors;
- It is grounded in processes of individual, team, and system adaptation, enhancement and reorganisation – which represent processes that underpin learning, growth, development and recovery;
- It indicates that diversity, coordination and collaboration are key elements of the processes that underpin capabilities for resilience; and
- It is open-ended enough to accommodate the application of diverse concepts and mechanisms from different literature, and support diverse methods and approaches to research.

Instead of formulating a comprehensive definition that accommodates the total variety of our foundational understanding, we have developed a concise definition that should be more accessible to a diverse range of stakeholders, but which still grasps the key constructs of our conceptualisation of resilience in healthcare. In the RiH program, we define resilience in healthcare as:

*… the capacity to adapt to challenges and changes at different system levels, to maintain high quality care.*

### Core questions to guide resilience in healthcare research: what to define, what to describe

A set of core questions need to be addressed to define the phenomenon of resilience, and to operationalise and apply the idea of resilience in healthcare research. These questions are referred to here as: resilience *for* what, *to* what, *of* what, and *through* what*.* These represent important elements for defining and researching resilience. Empirically exploring each of these questions is intended to provide detailed information about four key aspects of the phenomena of resilience: the purpose of resilience (for what), what triggers resilience (to what), what resources are involved (of what) and what activities and interaction are required for resilient performance (through what). It is relatively rare for research on resilience in healthcare to systematically operationalize resilience in such a tangible way, and we, therefore, advocate for using these questions to guide resilience research and to produce investigations of resilience that are more readily comparable. In the following we provide details on each question and how and why they are core to research.

#### Resilience for what?

What is the purpose of resilience? This depends on the nature, goals and objectives of the system being studied. Resilience concerns an emergent capacity of a system, but not the goal of that system [26]. Therefore, resilience should not be thought of as inherently ‘good’ or ‘bad’. This perspective contrasts some of the literature, which conflates ‘safety’ as generally being a good thing, with ‘resilience’; or which seeks resilience as an ultimate and overriding goal for any system. For example, systems that produce very poor care outcomes may be highly resilient and stable, and resistant to efforts to change, improve or disrupt them. The overall purpose and goal of a system is, therefore, different from how resilient that system is. Another way of stating this is that resilience is a secondary property of a system. Resilience does not define what a system does or seeks to achieve. A healthcare system’s primary objective is not simply to be resilient. A health system’s primary objective should be (for example) to deliver high quality healthcare to a population. Achieving that primary objective likely depends heavily on the healthcare system’s capacity to be resilient. If high quality care breaks down easily at the first sign of disruption, then that healthcare system cannot reasonably claim to be able to provide high quality healthcare.

*Therefore, it is important to articulate and define what resilience is for – what goals and objectives it is supporting – in any system or research study.*

#### Resilience to what?

To what should a healthcare system be resilient? What activates and triggers resilience? A system may have latent capacities and capability for resilience, but it is key to understand what, precisely, brings those capabilities into action. In much of the literature on resilience this relates to some adverse stress, disruption or surprise. Resilience has often been defined as the ability to respond to and deal with unanticipated and adverse events: failures, disruptions, errors and crises. This can be broadened to also consider resilience as the capability to respond to changes and challenges that imply more positive influence (e.g. the introduction of new technologies, the design of new work systems; the discovery of new medicines or products; or the identification of new best practices), which is consistent with recent development in the Safety II literature [25, 26]. This broader view, encompassing both the ‘dark’ and ‘light’ side of healthcare, implies that systems have resilient capacity if they can productively respond to and adapt to events that provide challenge or change in some way. For high quality healthcare systems, this responsiveness might represent the capacity to incorporate and spread new and productive ways of working across a healthcare system, as well as adapting to and learning from adverse events and other disruptions to the quality of care. The RiH program advocates that resilience research in healthcare needs to integrate both what goes well and what goes wrong as fundamental in resilience.

*Therefore, it is important to articulate and define what systems are resilient to – what triggers and activates resilience – in any system or research study.*

#### Resilience of what?

What parts, components, resources or participants are engaged in supporting or producing resilience? What are they, where are they located, and how do they relate to each other in the system? Researching resilience involves investigating into how resilience represents a set of human and organisational resources or activities coming together or being used in some way to address a problem, support learning or develop some new adaptation or approach to delivering healthcare [3, 26]. A key question, therefore, refers to what elements, actors or components are enrolled in this process – what are the materials of resilience within a particular system? This might include physical resources (e.g. equipment); financial resources (e.g. funding); human resources (e.g. personnel); cognitive resources (e.g. creativity, expert competence); emotional resources (e.g. empathy); epistemic resources (e.g. knowledge and ideas); informational resources (e.g. data and computational tools). Any of these things, and much more, might be relevant to explain and understand how resilience is supported in a system.

*Therefore, it is important to articulate and define what systems are using to enact resilience – what materials and resources underpin resilience – in any system or research study.*

#### Resilience through what?

What are the processes and mechanisms through which resilience is enacted or created? What forms of activities or modes of behaviour allow a system to effectively respond, adapt or learn? How resilience unfolds can take a wide variety of forms. Understanding the nature and the evolution of these processes is key to understanding the nature of resilience. Key processes are likely to include activities of engagement and communication amongst diverse stakeholders; activities of collaboration and coproduction through participative work; activities of learning and development through reflexive practice; and activities of decision-making, reorganisation, and reformation through systems design and change.

*Therefore, it is important to articulate and define the processes through which systems are able to be resilient – the mechanisms, activities and interactions that support resilience – in any system or research study.*

#### Added value of addressing the core questions for resilience research

Answering these four seemingly simple questions can help to provide a clear content of the phenomenon of resilience that is being investigated in any particular area of research work. In the future, we expect that addressing these questions could be assisted by drawing on a broad conceptual ‘menu’ that provides core categories and concepts of resilience that are applicable to a wide range of settings and studies to help provide a common language to describe and explain resilience.

## Conclusion

In this paper we have presented our definition of resilience and the boundaries and operational concepts of resilience in the Resilience in Healthcare Research Program. We define resilience as “the capacity to adapt to challenges and changes at different system levels, to maintain high quality care”. Our intent is that this concise definition of resilience will allow researchers, patients, practitioners, and all relevant stakeholders to engage with this important concept. In addition, we have suggested four core questions that can guide empirical approaches to resilience research. These questions can help researchers focus their future activities in this field, no matter what kind of system component or level that is under investigation. Conducting rigorous empirical work on resilience in all its forms, as well as actively engaging with stakeholders across the diverse landscape of healthcare will be fundamental to reforming the understanding of quality in current healthcare systems by developing an empirically-grounded and theoretically-informed approach to resilience in healthcare.

## Data Availability

Not applicable
